# Potential of Nerve Growth Factor (NGF)- and Brain-Derived Neurotrophic Factor (BDNF)-Targeted Gene Therapy for Alzheimer’s Disease: A Narrative Review

**DOI:** 10.7759/cureus.85814

**Published:** 2025-06-11

**Authors:** Prakhya Chowdary Koya, Saieesha Chowdary Kolla, Varun Madala, Suresh Babu Sayana

**Affiliations:** 1 Biological Sciences, Thomas Jefferson High School for Science and Technology, Alexandria, USA; 2 Department of Pharmacology, Mamata Medical College, Khammam, IND; 3 Department of Internal Medicine, All India Institute of Medical Sciences, Mangalagiri, Vijayawada, IND; 4 Department of Pharmacology, Government Medical College and General Hospital, Bhadradri Kothagudem, IND

**Keywords:** alzheimer’s disease, brain-derived neurotrophic factor (bdnf), cholinergic neurons, gene therapy, nerve growth factor (ngf), neuroprotection, synaptic plasticity

## Abstract

Alzheimer’s disease (AD) involves progressive degeneration of cholinergic and synaptic networks, leading to cognitive decline. Nerve growth factor (NGF) and brain-derived neurotrophic factor (BDNF), essential for neuronal survival and plasticity, have gained therapeutic interest. Particularly through gene therapy approaches. Preclinical studies have shown improved neuronal integrity and memory restoration. Nonetheless, clinical translation faces significant challenges, including invasive delivery, vector limitations, and receptor dysregulation. Gene therapy must be focused on precision-targeted, minimally invasive delivery systems, controlled gene expression, and early-stage intervention. This review critically evaluates NGF- and BDNF-based gene therapies, highlighting advances, limitations, and future directions that may position neurotrophin modulation as a viable disease-modifying strategy in AD.

## Introduction and background

Alzheimer’s Disease (AD) is a neurodegenerative disorder. Progressive cognitive decline, memory impairment, and the degeneration of neurons, particularly in the hippocampus and cortex, are the major disease aspects seen in AD. Loss of cholinergic function plays a pivotal role in synaptic plasticity and memory processes in AD pathophysiology [1-4]. Basal forebrain cholinergic neurons (BFCNs) are one of the most severely affected groups of neurons that project to key brain regions involved in learning and memory, such as the cortex and hippocampus [5]. The nucleus basalis of Meynert (nbM) is the primary source of these cholinergic projections, and it is one of the primary regions to exhibit degeneration in AD. A significant decline in cognitive function also results from degeneration of the nbM [6]. Nerve growth factor (NGF) and brain-derived neurotrophic factor (BDNF) have garnered attention as potential therapeutic agents because both NGF and BDNF are critical for the development, survival, and maintenance of neurons in the brain, and are believed to contribute to the neurodegenerative process in AD [7]. Thus, NGF has long been associated with the survival and trophic maintenance of BFCNs, which are among the earliest affected in AD [1-4]. Likewise, BDNF plays a key role in synaptic plasticity, learning, and memory, particularly within the hippocampus and cortex regions heavily compromised in AD [7,8]. Preclinical studies have shown that NGF gene delivery can prevent cholinergic degeneration and enhance cognitive performance in rodent and primate models [9,10], while BDNF supplementation has been shown to restore synaptic integrity and memory function independently of amyloid clearance [11-13]. Given this biological significance, gene therapy strategies aimed at delivering NGF and BDNF to vulnerable brain regions have emerged as a promising disease-modifying approach in AD.

Biological rationale and pathophysiology

NGF-based gene therapy is grounded in its well-documented trophic support for cholinergic neurons. Despite this, studies over the last two decades have revealed an important paradox in AD: while NGF levels in the brain are stable or even elevated, the neurodegeneration of cholinergic neurons persists. This has been attributed to a breakdown in the NGF metabolic pathway, specifically the impaired conversion of pro-NGF to its mature form (mNGF), which limits its neuroprotective effects. Similarly, BDNF has been shown to have a critical role in synaptic function and neuroplasticity, and its reduced levels are associated with cognitive decline in AD. The decay of both NGF and BDNF in AD brains has been attributed to receptor downregulation (tropomyosin receptor kinase A (TrkA) for NGF and tropomyosin receptor kinase B (TrkB) for BDNF). It also impaired transport mechanisms and altered intracellular signaling pathways. Therefore, gene therapy targeting these pathways holds great potential for restoring neuronal function and slowing disease progression [1].

Therapeutic potential of NGF and BDNF

The potential of NGF-based gene therapy lies in its ability to restore cholinergic function, particularly through the rescue of BFCNs in AD [4]. Preclinical studies in rodent and primate models (AD models) using viral vector-mediated NGF delivery have demonstrated substantial improvements in neuronal survival, axonal sprouting, and spatial memory [9,10]. Both NGF and BDNF are pivotal neurotrophins with well-established roles in neuronal survival and plasticity. NGF supports basal forebrain cholinergic neurons, which are early targets in AD pathology and gene therapy trials have shown its long-term bioactivity and safety in vivo. In a phase 1 trial, adeno-associated virus serotype 2 (AAV2)-NGF delivery led to persistent expression of NGF and axonal sprouting toward the infusion site, without adverse pathological findings. Similarly, BDNF has demonstrated efficacy in restoring synaptic function and memory in preclinical AD models, even without affecting amyloid levels. These findings provide convincing translational rationale for exploring NGF and BDNF gene delivery as disease-modifying interventions in AD [3,4,9,10,14].

Similarly, BDNF-based gene therapy has shown promise in preclinical studies, in which direct delivery of BDNF into the hippocampus and cortex improved synaptic density and cognitive performance in AD models. BDNF’s potential is to promote neurogenesis and synaptic plasticity, which are critical for restoring cognitive function in AD [15]. Studies have also suggested that BDNF supplementation may provide neuroprotection even in the presence of amyloid plaques. Therefore, offers a potential therapeutic avenue for patients where anti-amyloid therapies have not proven effective [11-13].

The delivery of NGF and BDNF to the brain is one of the key challenges in gene therapy for AD [16]. Traditional delivery methods, such as intracerebral injections or intravenous infusions, have been mostly invasive. These often fail to achieve sufficient targeting of the area with accuracy. To overcome these challenges, various advanced gene delivery technologies have been developed, including viral vectors, non-viral systems, and stem cell-based strategies. However, each of these methods still faces hurdles such as immune response, vector spread, and issues in blood-brain barrier crossing. The main objective of this review is to analyze and compare NGF and BDNF targeted gene therapy approaches, discuss delivery vectors, efficacy, safety, and propose a roadmap for combinatorial approaches [11,13,17-19].

## Review

Database search strategy and selection

Relevant peer-reviewed articles and clinical guidelines (between 2000 and 2025) were searched in databases from PubMed, Scopus, Web of Science, and Google Scholar. Key word included are: ("Alzheimer's disease" OR dementia) AND ("viral" OR "non-viral vectors" OR "gene therapy" OR "gene transfer" OR "viral vector" OR "AAV" OR "lentivirus" OR "CRISPR") AND ("Neurotrophic factors" OR "NTF" OR "NGF" OR "nerve growth factor" OR "BDNF" OR "brain-derived neurotrophic factor"). Human/Animal studies on AD, studies using NGF or BDNF gene delivery were included in this narrative review. 

NGF-based gene therapy

NGF is a prototypical neurotrophin critical for the development, maintenance, and plasticity of BFCNs. BFCNs are among the earliest and most selectively affected neuronal populations in AD. The rationale for NGF-based gene therapy stems from its well-characterised trophic support to these neurons [1-3]. A substantial body of work has sought to harness the neuroprotective effects of NGF via gene therapy platforms, ranging from viral vector-mediated delivery to encapsulated cell bio-delivery devices [3,9,10,14,20,21].

Biological Rationale and Early Insights

Foundational studies from the 1990s and early 2000s, such as those by Scott et al. (1994) and Counts and Mufson (2005), established that despite stable or even elevated NGF levels in the AD brain, cholinergic neurodegeneration persisted, suggesting a disruption in NGF processing or signalling pathways [21,22]. This apparent contradiction was later clarified by the discovery that the conversion of proNGF to mature NGF (mNGF) was severely impaired, and its degradation was accelerated [1].

Preclinical Success and Translational Advances

Animal studies provided robust proof of concept that NGF gene therapy could rescue cholinergic neurons from lesion-induced or age-related degeneration. Tuszynski and Blesch (2004), followed by Rafii and colleagues (2014), used AAV2 vectors to deliver NGF directly to the nbM in rodent and primate models. These studies showed not only survival of BFCNs but also axonal sprouting and improved spatial memory [3,10]. Lentiviral approaches and encapsulated cell implants, those evaluated by Eriksdotter and colleagues, also yielded neurotrophic effects without eliciting significant immune responses. These encouraging results propelled NGF into early clinical trials [18,23]. A landmark Phase I study by Tuszynski et al. (2015) involving eight AD patients demonstrated the feasibility of both ex vivo fibroblast-based and in vivo AAV2-mediated NGF gene delivery. Postmortem analysis revealed persistent NGF expression, cell hypertrophy, and axonal sprouting in the targeted nbM region. Further, this study provided a strong histopathological validation [14].

Clinical Trials and Outcomes

The Phase II randomized controlled trial by Rafii et al. (2018) using AAV2-NGF (CERE-110) failed to produce statistically significant improvements in cognitive endpoints such as Alzheimer’s Disease Assessment Scale-Cognitive Subscale (ADAS-Cog) or Mini-Mental State Examination (MMSE) over a 24-month period. Nonetheless, the procedure was deemed safe and well-tolerated, offering valuable lessons in neurosurgical vector delivery [9]. Postmortem evaluation of subjects from the trial, reported by Castle et al. (2020), provided a critical insight that the radial diffusion of AAV2 was found insufficient to reach critical neuronal populations, citing the importance of delivery precision and vector kinetics in determining therapeutic success [20].

One of the most formidable challenges in NGF-based gene therapy is effective delivery to deep brain structures. Intracerebral injection, though precise, is invasive and limits scalability. AAV2’s poor parenchymal spread needs either multiple injection sites or innovations such as convection-enhanced delivery (CED). The need for real-time imaging, such as MRI-guided delivery with gadolinium-labeled agents, has been proposed to improve targeting [3,18,24-26]. Non-invasive approaches are also under investigation. Sasmita (2019) reviewed intranasal delivery and stem cell-based NGF secretion, while Mitra et al. (2019) highlighted encapsulated cell biodelivery (ECB) as a promising alternative, capable of providing localized, regulated NGF release without direct genetic manipulation. However, these systems still face challenges such as inflammatory encapsulation and variability in protein output over time [17,18].

Recent literature has pushed the field toward greater molecular precision. Mufson et al. (2019) and Do Carmo et al. (2022) emphasized the imbalance between pro-apoptotic p75 Neurotrophin Receptor (p75NTR) signaling and reduced TrkA receptor expression as a crucial determinant of cholinergic vulnerability in AD. Thus, therapies that merely increase total NGF may be insufficient unless they restore TrkA signaling or mitigate p75-mediated toxicity [24,26].

Moreover, emerging concepts from Chen and Mobley (2019) introduced the “signalling endosome hypothesis,” which posits that deficits in NGF-containing endosome transport, rather than NGF itself, precipitate neuronal atrophy. This shift has inspired investigations into endosome-targeted interventions and Rab5-modulating strategies [25]. A comparative overview of NGF-based gene therapy studies is discussed in Table 1.

**Table 1:** Comparative Overview of NGF-Based Gene Therapy Studies in AD. This table summarises key preclinical and clinical studies investigating NGF-based gene therapy for AD. Each row presents the study reference, model or subject population, the method of NGF delivery (e.g., viral vectors, encapsulated cells), main findings related to therapeutic efficacy or biological response, and the principal limitations noted in each study. AAV2: Adeno-Associated Virus serotype 2, AD: Alzheimer’s Disease, ECB: Encapsulated Cell Biodelivery, MCI: Mild Cognitive Impairment, MMSE: Mini-Mental State Examination, nbM: Nucleus Basalis of Meynert, NCI: No Cognitive Impairment, NGF: Nerve Growth Factor, PET: Positron Emission Tomography, p75NTR: p75 Neurotrophin Receptor, ROS: Religious Orders Study, TrkA: Tropomyosin receptor kinase A

Study / Trial	Model / Subjects	Delivery Method	Key Findings	Limitations	Summary of delivery methods
Tuszynski & Blesch (2004) [3]	Phase I clinical trial of NGF gene therapy in AD. 8 patients with early-stage AD Model: Human clinical trial	Ex vivo gene therapy using autologous fibroblasts genetically modified to secrete human NGF implanted into the nbM	NGF gene therapy showed promise in preclinical models (rodent and primate) for preventing cholinergic neuronal degeneration. In animal studies, NGF gene delivery prevented neuronal loss, reversed age-related atrophy, and restored cholinergic function. No significant adverse effects were reported in preclinical safety studies, and NGF expression persisted for over a year in animal models.	Potential risks include the unwanted spread of NGF to non-targeted brain regions, leading to side effects such as pain and weight loss. The trial's focus is on safety, and the efficacy of NGF gene therapy in slowing AD progression will require further investigation in Phase II trials.	Stereotactic injection enabled precise NGF delivery in both ex vivo and in vivo trials, minimizing off-target effects. Intraventricular infusion caused adverse effects from widespread exposure. CED was noted but not used. AAV-based delivery proved the safest and most sustained method.
Rafii et al. (2014, Phase 1) [10]	10 participants with mild to moderate AD	Stereotactic injection of AAV2-NGF vector into the nbM	AAV2-NGF was well-tolerated for 2 years. No accelerated cognitive decline in PET scans or neuropsychological tests. Long-term NGF expression confirmed at autopsy.	Small sample size, limiting efficacy conclusions. Lack of control group, making efficacy interpretation difficult.	Stereotactic injection enabled precise AAV2-NGF delivery to the NBM, guided by intraoperative MRI for accuracy. Ex vivo NGF delivery used similar targeting but showed variable cell survival. CED was not used but noted as a future option.
Rafii et al. (2018, Phase 2) [9]	Phase 2 randomised clinical trial of AAV2-NGF gene therapy for AD. 49 participants with mild to moderate AD	Stereotactic intracerebral injections of AAV2-NGF into the nbM	AAV2-NGF was safe and well-tolerated. No significant difference in cognitive decline between treatment and placebo groups. Secondary outcomes showed trends toward worsening in the treatment group.	Small sample size, limiting power to detect efficacy. No amyloid PET imaging or cerebrospinal fluid analysis to rule out non-AD diagnoses. No clear efficacy on cognitive outcomes or biomarkers.	Stereotactic AAV2-NGF injection enabled precise, localized delivery to the basal forebrain with MRI-guided targeting; CED was not used but noted as a potential method. Postmortem histology confirmed accurate placement and restricted NGF expression.
Tuszynski et al. (2015) [14]	NGF Gene Therapy for AD. Postmortem study. 10 patients with early AD	Ex vivo and in vivo NGF gene transfer using viral vectors AAV2 or autologous fibroblasts	NGF induced trophic responses, including axonal sprouting and neuronal hypertrophy. NGF expression persisted for up to 10 years after treatment. No adverse pathological effects related to NGF were observed.	Limited sample size (10 patients). NGF gene transfer effects may vary over time. No control group in postmortem study for long-term effects.	Stereotactic AAV2 delivery under imaging guidance achieved safe, localized, and reproducible gene expression.
Castle et al. (2020) [20]	Postmortem analysis in a clinical trial of AAV2-NGF gene therapy for AD. 3 deceased patients from the Phase 1 AAV2-NGF clinical trial	Stereotactic injection of AAV2-NGF into the nbM	NGF expression persisted for more than 7 years at injection sites. No significant cognitive decline between treatment and placebo groups in the Phase 2 trial. Limited spread of AAV2-NGF from the injection sites.	Inaccurate stereotactic targeting of some injection sites. Lack of significant cognitive improvement despite NGF expression.	stereotactic AAV2 delivery with imaging support ensured high delivery accuracy and biological specificity.
Mitra et al. (2019) [18]	Innovative Therapy for AD. Focus on NGF Biodelivery.	Biodelivery of NGF using ECB	Successful NGF delivery to the basal forebrain of AD patients. Improved cholinergic markers and cognitive functions in some patients. Safety and tolerability were demonstrated.	Variations in NGF release across patients. Issues with cell survival in certain implants. Surgical complications and potential inflammation at implant sites.	Precision delivery via stereotactic AAV2 injection with imaging guidance proved effective and reproducible in targeting deep brain regions.
Counts & Mufson (2005) [21]	NGF Receptor Alterations in AD and Mild Cognitive Impairment. Subjects from the ROS with MCI, mild AD, and NCI	postmortem brain tissue analysis	In MCI and mild AD, NGF receptors TrkA and p75NTR are reduced by ~50%. ProNGF levels increase by 40-60% in cortical regions during MCI and AD. TrkA reduction correlates with cognitive decline (MMSE scores). NGF receptor downregulation suggests a phenotypic silencing during early AD stages.	Observations are based on postmortem tissue; results may not fully represent in vivo changes.	MRI-guided stereotactic AAV2 delivery ensured precise, safe, and reproducible gene targeting in deep brain structures.

Future Directions and Combinatorial Models

The cumulative findings suggest that NGF gene therapy is biologically viable and clinically safe, but faces substantial translational bottlenecks, most notably delivery inefficiency, receptor imbalance, and irreversibility of advanced-stage disease [27]. Future therapeutic models may benefit from combinatorial gene therapy, co-delivering NGF with other agents such as BDNF, neprilysin, or anti-inflammatory cytokines. Tang et al. (2025) and Revilla et al. (2014) provide encouraging preclinical data supporting multi-modal neurotrophic support [13,28]. Additionally, personalized medicine approaches like induced pluripotent stem cells (iPSC)-derived NGF-secreting grafts or vector optimization for APOE4 carriers are beginning to reshape the landscape. The emphasis is shifting toward early intervention, possibly even at the prodromal or mild cognitive impairment (MCI) stage, where NGF support could stabilize synaptic function and delay degeneration [14,29].

NGF-based gene therapy represents one of the most enduring and scientifically grounded strategies for disease modification in AD [30]. While the journey from molecular rationale to clinical efficacy remains incomplete. Recent mechanistic insights and technological advances continue to refine its potential. Moving forward, precision delivery, receptor-targeted modulation, and combinatory strategies will be pivotal in realizing NGF’s promise as a cornerstone of next-generation AD therapeutics [31,32].

BDNF-based gene therapy

BDNF has emerged as a critical molecular target in the pursuit of disease-modifying treatments for AD. As the most abundant neurotrophin in the adult brain, BDNF supports the survival, differentiation, and synaptic plasticity of cortical and hippocampal neurons. The decline of BDNF expression in early and progressive stages of AD correlates with impaired long-term potentiation, memory loss, and reduced cognitive resilience. Restoring BDNF signalling through gene therapy has garnered increasing attention as a viable therapeutic strategy, either as a standalone approach or in tandem with other disease-modifying agents [31].

Biological Basis and Pathological Relevance

BDNF exerts its effects primarily through binding to its high-affinity receptor tropomyosin receptor kinase B (TrkB), initiating intracellular cascades that promote synaptic growth, plasticity, and thereby resilience against neurotoxicity. In AD, BDNF levels are reduced in the hippocampus and temporal cortex, and TrkB receptor expression is often downregulated or mislocalized, disrupting downstream ERK, PI3K/Akt, and cAMP response element-binding protein (CREB) pathways [8,33]. Iwasaki et al. (2012) demonstrated that direct delivery of BDNF via Sendai virus vectors to the hippocampus of AD model mice restored synaptic density and enhanced memory retention, even without altering amyloid burden. This finding supports a model where BDNF supplementation may confer neuroprotective benefits independent of classical amyloid or tau pathologies [11].

Moreover, transcriptional dysregulation of BDNF expression, often linked to impaired CREB phosphorylation. This phenomenon has been identified as an early molecular deficit in AD. Caccamo et al. (2010) showed that lentiviral delivery of CREB-binding protein (CBP) into the hippocampus not only restored CREB function but also increased BDNF levels, rescuing spatial learning deficits in 3xTg-AD mice [12]. Such results suggest that enhancing the transcriptional machinery upstream of BDNF may serve as an effective gene therapy proxy for directly boosting its expression.

Gene Therapy Vectors and Delivery Platforms

Multiple vector systems have been explored for BDNF delivery, including AAVs, lentiviruses, and non-viral vectors. Each offers distinct advantages and limitations. AAV-based constructs have demonstrated robust transgene expression with minimal immune response; however, their packaging limitations and need for intracerebral injection remain significant barriers. Iwasaki’s SeV-BDNF vector offered non-integrative gene delivery with high expression levels, a useful feature for transient, regulated treatment strategies [12,13,19,34].

Recently, Tang et al. (2025) employed a recombinant adeno-associated vector (AAVT42-BDNF) in an amyloidogenic AD mouse model. Same reported restored hippocampal BDNF levels alongside significant cognitive improvement, without any reduction in amyloid beta (Aβ) plaques. Such findings reinforce the hypothesis that synaptic restoration alone may yield functional benefits, even when core pathological burdens persist. However, it remains to be seen whether this functional improvement translates into long-term disease modification in human subjects [13]. In parallel, stem cell-based delivery systems are gaining momentum. The 2015 study in Stem Cell Reports used neural stem cells (NSCs) overexpressing BDNF in a model of dementia with Lewy bodies. The study has demonstrated improved motor and cognitive performance via BDNF-dependent restoration of dopaminergic and glutamatergic homeostasis. The disease model focused on α-synuclein pathology, the mechanistic pathway involving BDNF aligns closely with hippocampal vulnerability observed in AD [19].

Synaptic and Cognitive Restoration

BDNF’s role in cognitive enhancement stems from its ability to promote synaptogenesis, dendritic branching, and neurogenesis, particularly in the dentate gyrus. These synaptic and structural enhancements are consistently associated with improvements in behavioural outcomes across AD models. For example, in 3xTg-AD and APP/PS1 mice, BDNF restoration has been shown to improve performance in the Morris Water Maze and Novel Object Recognition tests, indicating enhanced spatial learning and memory. Such findings underscore the translational potential of BDNF-targeted therapies for restoring cognitive function in AD. Restoration of BDNF in 3xTg-AD and other transgenic models has consistently led to increased expression of N-methyl-D-aspartate (NMDA) receptor subunits (NR2A, NR2B), synapsin, and PSD-95, all of which are critical for long-term potentiation. Furthermore, BDNF signaling also indirectly supports cholinergic integrity, making it a convergent point for multiple neurotransmitter systems impacted in AD [11,13,19,28,35].

A compelling aspect of BDNF gene therapy is its capacity to reverse cognitive impairment in the absence of amyloid or tau reduction. This has important implications for patients in whom anti-amyloid strategies have proven ineffective. Caccamo et al.’s CBP study, for example, found no significant change in Aβ or phosphorylated tau levels, yet observed marked improvements in learning and memory performance, likely by BDNF-mediated circuit repair [12,35].

Safety Considerations and Therapeutic Limitations

Despite its promise, BDNF-based gene therapy faces several translational obstacles. BDNF is a pleiotropic molecule with widespread effects, and unregulated expression may lead to adverse outcomes such as seizures, pain sensitization, and aberrant synaptic remodelling. Additionally, isoform-specific effects (e.g., pro-BDNF vs. mature BDNF) need careful consideration, as pro-BDNF can paradoxically promote synaptic pruning via p75NTR [11,19,28,34]. Vector delivery poses another challenge. Most preclinical studies rely on direct intracranial injection, an approach not easily translatable to routine clinical care [36]. A comparative summary of BDNF-based gene therapy approaches in AD and related models is discussed in Table 2.

**Table 2:** Comparative Summary of BDNF-Based Gene Therapy Approaches in AD and Related Models. This table summarizes key studies on BDNF gene therapy in Alzheimer’s disease models, highlighting the delivery methods, primary findings, and major limitations. It reflects diverse approaches including viral vectors, transcriptional enhancers, and stem cell carriers aimed at restoring synaptic function and cognitive performance. 3xTg-AD: Triple Transgenic Mouse Model of Alzheimer’s Disease, AAV: Adeno-Associated Virus, AAVT42: Adeno-Associated Virus serotype T42, AD: Alzheimer’s Disease, Aβ: Amyloid Beta, ASO: α-Synuclein Overexpression, BDNF: Brain-Derived Neurotrophic Factor, CBP: CREB-Binding Protein, CREB: cAMP Response Element-Binding Protein, GDNF: Glial cell line-Derived Neurotrophic Factor, MC65: Human neuroblastoma cell line (expressing APP-C99 upon tetracycline withdrawal), NF-α1: Neurotrophic Factor Alpha 1, NMDA: N-Methyl-D-Aspartate, NSC: Neural Stem Cell, SeV: Sendai Virus

Study / Trial	Model / Subjects	Delivery Method	Key Findings	Limitations
Iwasaki et al. (2012) [11]	Sendai Virus Vector-Mediated BDNF Expression Ameliorates Memory Deficits in AD. Tg2576 transgenic mouse model of AD.	SeV/BDNF vector injection into the alveus hippocampi (subcortical white matter)	SeV/BDNF successfully induced BDNF expression in hippocampal neurons. Amelioration of synaptic degeneration and memory deficits in Tg2576 mice. BDNF induction preserved both pre- and postsynaptic structures.	BDNF induction was transient, with effects decreasing over time. No significant changes in amyloid plaque burden were observed. The study only tested early-stage intervention; long-term effects were not evaluated.
Caccamo et al. (2010) [12]	CBP Gene Transfer Increases BDNF Levels and Ameliorates Learning and Memory Deficits in AD. 3xTg-AD transgenic mice model of AD.	Lentiviral delivery of CBP to the lateral ventricle	CBP overexpression in the hippocampus restored CREB phosphorylation. Improved learning and memory deficits in 3xTg-AD mice without affecting Aβ or tau pathology. Increased BDNF levels are linked to improved cognitive function and NMDA signalling.	No changes observed in Aβ or tau pathology despite memory improvements. Effects of CBP gene transfer have not been assessed long-term.
Tang et al. (2025) [13]	Hippocampus-targeted BDNF gene therapy to rescue cognitive impairments of AD in multiple mouse models. APP/PS1, rTg4510, 3 × Tg AD mouse models and adult male Cynomolgus macaque.	AAVT42 virus-mediated BDNF gene delivery to the hippocampus	AAVT42 efficiently transduced neurons, improving cognitive functions in AD mouse models. Long-term BDNF expression alleviated cognitive impairments without affecting amyloid or tau pathology. Gene expression profiling indicated upregulation of synaptic genes, supporting neuroprotective effects.	BDNF treatment did not affect amyloid plaque or tau phosphorylation. The therapeutic effect of BDNF was partially model-dependent and did not fully reverse memory deficits in some models.
N. R. S. Goldberg et al. (2015) [19]	Neural Stem Cells Rescue Cognitive and Motor Dysfunction in a Transgenic Model of Dementia with Lewy Bodies. Transgenic mice with α-synuclein overexpression	Stereotaxic injection of human-NSCs into the striatum	NSC transplantation significantly improved cognitive and motor function in ASO mice. BDNF expression from NSCs played a critical role in recovery. NSCs enhanced dopaminergic and glutamatergic systems.	No effect on α-synuclein pathology (Lewy body-like inclusions). NSC migration did not reach the hippocampus. The study did not evaluate the long-term effects of NSC transplantation.
Shaikh et al. (2025) [34]	Neurotrophic Factor Alpha 1 Gene Therapy in AD. Transgenic 3xTg-AD mice model of Alzheimer's disease	Adeno-AAV vector delivery to the hippocampus	NF-α1 gene delivery improved cognitive function and reduced amyloid plaques in AD mice. NF-α1 gene therapy promoted neurogenesis and improved hippocampal synaptic integrity. Significant reduction in tau hyperphosphorylation and neuroinflammation.	No long-term human clinical data available for NF-α1 gene therapy. Only tested in transgenic mouse models, not in human trials.
Revilla et al. (2014) [28]	Lenti-GDNF Gene Therapy Protects Against AD-Like Neuropathology. 3xTg-AD mice model and MC65 human neuroblastoma cells	Lentiviral vector-mediated overexpression of GDNF in hippocampal astrocytes	GDNF gene therapy preserved learning and memory in 3xTg-AD mice. GDNF therapy did not significantly reduce amyloid and tau pathology but induced upregulation of BDNF. In MC65 cells, GDNF reduced oxidative stress and cell death.	Limited reduction of amyloid and tau pathologies despite cognitive improvements. Short duration of therapy (6 months). Only tested in animal and cell models, with no human clinical data.

Combinatorial Approaches and Future Outlook

Given the complexity of AD, future strategies are likely to adopt a combinatorial approach, coupling BDNF gene therapy with agents that address complementary pathways, such as anti-amyloid antibodies, NGF support for cholinergic neurons, or anti-inflammatory cytokines [37]. There is also growing interest in co-targeting CREB and BDNF through epigenetic or transcriptional enhancers to potentiate endogenous neurotrophic support. The application of iPSCs genetically engineered to secrete BDNF is another promising direction. These personalized cell lines could provide long-term trophic support without the need for repeated invasive delivery [38].

BDNF-based gene therapy presents a strategy for restoring synaptic function and mitigating cognitive decline in AD. BDNF is positioned as a central candidate in next-generation therapeutics. However, for this potential to be fully realized, significant advances are needed in delivery technologies, isoform-specific modulation, and early diagnostic precision. As part of a broader multimodal framework, BDNF gene therapy may ultimately offer a durable benefit in both monogenic and sporadic forms of AD [39].

Limitations

Both NGF- and BDNF-based gene therapies face significant translational challenges, despite convincing biological rationale and encouraging preclinical outcomes. A primary limitation common to both is the invasive nature of delivery, typically requiring stereotactic intracerebral injections to reach deep brain structures such as the nbM for NGF and the hippocampus for BDNF. These surgical approaches are not readily scalable or feasible for routine clinical use in large patient populations.

For NGF therapy, targeting accuracy remains a critical concern. As evidenced in the AAV2-NGF trials (Rafii et al., 2018; Castle et al., 2020), even modest deviations in vector placement can result in suboptimal cholinergic engagement, and it may further result in therapeutic failure [9,20]. Additionally, NGF metabolic dysregulation involving impaired proNGF conversion and increased degradation by MMPs suggests that simply increasing NGF levels may be insufficient unless accompanied by enzymatic pathway correction [1,24].

In the case of BDNF, while its broad neurotrophic activity is advantageous, it also often presents with risks. Unregulated or ectopic BDNF expression may lead to adverse effects such as heightened excitability, pain sensitisation, or seizure susceptibility. Moreover, isoform-specific effects (e.g., pro-BDNF acting through p75NTR to mediate synaptic pruning) necessitate precise control over post-translational processing. It is an area still poorly understood in clinical contexts. Both neurotrophins are also vulnerable to age-related receptor downregulation. Expression of TrkA (NGF receptor) and TrkB (BDNF receptor) declines, reducing the efficacy window for therapy in some advanced AD cases. Additionally, endosomal trafficking deficits, especially in NGF signalling and can compromise intracellular signalling cascades even if extracellular delivery is successful [25]. From a broader perspective, neither NGF nor BDNF therapies have yet demonstrated consistent cognitive benefits in human trials. This has highlighted a translational gap that may be due to late-stage intervention, insufficient expression levels, or disease heterogeneity.

Future directions

Overcoming the current limitations in neurotrophin-based gene therapy for AD will require multifaceted innovation spanning molecular engineering, delivery technologies, and diagnostic integration. One of the most pressing needs is the development of less invasive, targeted delivery systems that can reliably bypass the blood-brain barrier. Emerging techniques such as focused ultrasound (currently in early-phase clinical trials), intranasal gene vectors (primarily validated in animal models), and nanoparticle-based carriers (under active preclinical investigation) offer non-invasive or minimally invasive alternatives to conventional stereotactic injection. Furthermore, real-time, image-guided neurosurgical approaches such as MRI-convection enhanced delivery (MRI-CED) are being refined in large animal studies and early clinical settings, showing promise for precise, spatially restricted administration of gene therapies with minimal collateral exposure. Equally important is the integration of controlled gene expression systems to improve therapeutic safety and efficacy. The use of inducible promoters, tissue-specific enhancers, and microRNA-responsive switches allows for spatiotemporal modulation of neurotrophin production, helping to fine-tune expression levels and reduce off-target effects. This precision control is especially critical in the delicate neural environment affected by AD pathology.

Given the multifactorial nature of AD, combining neurotrophins delivery with complementary strategies represents a compelling future direction. Gene therapy platforms co-delivering NGF or BDNF with anti-amyloid agents, tau-directed molecules, or anti-inflammatory cytokines could address parallel aspects of AD pathogenesis. Additionally, pairing neurotrophic support with synapse-regulating co-factors such as CREB or CBP may amplify downstream cognitive benefits and synaptic resilience. Another innovative approach involves modulation of the neurotrophin signalling pathway itself. Rather than direct delivery of NGF or BDNF, enhancing endogenous signalling through upregulation of CBP, activation of tissue plasminogen activator (tPA)/plasmin cascades, or inhibition of matrix metalloproteinase (MMP)-mediated degradation may offer more physiologically nuanced and sustainable interventions. This strategy is particularly relevant in cases where neurotrophin receptor integrity or retrograde transport mechanisms are impaired.

The timing of intervention is also pivotal. As receptor expression and neuronal plasticity decline during late-stage AD, future trials must prioritize early or prodromal phases, such as mild cognitive impairment. Integration of neurotrophin therapies with fluid-based or neuroimaging biomarkers can enable stratified, stage-specific treatment strategies, aligning with the principles of precision medicine. Finally, iPSC-based platforms are emerging as powerful tools for personalised and sustained neurotrophins delivery. Engineered iPSCs capable of secreting NGF or BDNF, particularly when combined with genome editing technologies like CRISPR-based transcriptional control, hold transformative potential for tailoring gene therapy to individual patient profiles while enabling long-term efficacy.

## Conclusions

Neurotrophin-based gene therapies that are centered on NGF and BDNF offer a fascinating neurobiological rationale for disease-modifying interventions in AD. The established roles of NGF in cholinergic neuronal maintenance and of BDNF in synaptic plasticity form the theoretical foundation for these approaches. Despite promising preclinical data demonstrating enhanced neuronal survival, axonal integrity, and cognitive restoration, translation into clinical efficacy has remained elusive. Major challenges include the invasive nature of intracerebral delivery, suboptimal vector diffusion, receptor downregulation, and the complexity of targeting structurally and functionally compromised brain regions. These limitations highlight a critical translational bottleneck and highlight the importance of precision-targeted delivery, optimized expression systems, and early-stage intervention in therapeutic design.

Future research must focus on a multifaceted framework incorporating non-invasive delivery technologies, inducible and tissue-specific expression constructs, and then combinatorial approaches targeting multiple pathological axes. Emerging strategies, including the use of iPSC-derived neurotrophin-secreting cells and gene editing tools such as CRISPR, offer potential to overcome current barriers while providing patient-specific therapeutic modulation. Furthermore, aligning these interventions with biomarker-driven stratification and prodromal stage implementation may enhance their disease-modifying capacity. While gene therapy for AD remains in its developmental infancy, rigorous mechanistic insight and technological innovation are poised to transform neurotrophin-based interventions into viable clinical modalities in the pursuit of halting or reversing neurodegenerative decline.
